# Association of Serial Lactate-to-Albumin and C-Reactive Protein-to-Albumin Ratios with In-Hospital Mortality After Out-of-Hospital Cardiac Arrest

**DOI:** 10.3390/jcm15134851

**Published:** 2026-06-23

**Authors:** Wan Young Heo, Dong Hun Lee, Seok Jin Ryu, Byung Kook Lee, Yong Hun Jung, Kyung Woon Jeung

**Affiliations:** Department of Emergency Medicine, Chonnam National University Hospital, Chonnam National University of Medical School, 42, Jebong-ro, Donggu, Gwangju 61469, Republic of Korea; bean3927@naver.com (W.Y.H.); samahalak@naver.com (S.J.R.); bbukkuk@hanmail.net (B.K.L.); xnxn77@hanmail.net (Y.H.J.); neoneti@hanmail.net (K.W.J.)

**Keywords:** cardiac arrest, lactate-to-albumin ratio, C-reactive protein-to-albumin ratio, mortality

## Abstract

**Background**: The lactate-to-albumin ratio (LAR) and C-reactive protein-to-albumin ratio (CAR) are biomarkers for metabolic stress and inflammation. However, their prognostic significance after return of spontaneous circulation (ROSC) in out-of-hospital cardiac arrest (OHCA) remains unclear. Therefore, this study aims to investigate the association between serial LAR/CAR measurements and in-hospital mortality. **Methods**: This retrospective observational cohort study included adult comatose patients with OHCA treated with targeted temperature management between January 2022 and December 2025. Serum lactate, albumin, and C-reactive protein levels were measured at admission and at 24, 48, and 72 h after ROSC. The primary outcome was in-hospital mortality. Multivariable logistic regression analyses were performed to assess independent associations of LAR and CAR with in-hospital mortality, and discriminatory performance was assessed using the area under the receiver operating characteristic curve (AUC). **Results**: Of the 284 eligible patients, 253 were included in the final analysis. Of these, 80 patients died in hospital, corresponding to an in-hospital mortality rate of 31.6%. LAR and CAR were significantly higher in non-survivors than in survivors at admission and at 24, 48, and 72 h after ROSC. After adjustment for potential confounders, LAR was associated with in-hospital mortality at all assessed time points. CAR was independently associated with in-hospital mortality at admission and at 48 and 72 h after ROSC, but not at 24 h. The AUCs of LAR for predicting in-hospital mortality ranged from 0.702 to 0.734, whereas those of CAR ranged from 0.640 to 0.690. **Conclusions**: In this single-center retrospective cohort of post-ROSC OHCA patients, sequential tracking of LAR and CAR profiles during the first 72 h after ROSC provided meaningful insights into in-hospital mortality. LAR showed a more consistent independent association with mortality and fair discriminatory performance, whereas CAR demonstrated limited prognostic value despite its association with mortality.

## 1. Introduction

Although return of spontaneous circulation (ROSC) can be achieved in some patients with out-of-hospital cardiac arrest (OHCA), mortality remains high, with survival declining to 29% at 24 h and 16% at 30 days. Among patients who survive the initial hospitalization, 10-year survival is only 62–64% [1,2]. This excess mortality is largely attributable to post-cardiac arrest syndrome, which includes post-cardiac arrest brain injury, myocardial dysfunction, systemic ischemia–reperfusion response, and persistent underlying pathology [3,4]. During the post-resuscitation period, multimodal prognostic indicators may support individualized treatment planning and prognostic counseling, including timely, structured communication with family members or surrogates [5,6]. Accordingly, numerous prognostic factors and risk models have been investigated [4,7].

Serum lactate reflects tissue perfusion adequacy and metabolic stress intensity (often paralleling shock severity) and is one of the most widely used biomarkers associated with poor outcomes in critically ill patients. In patients with OHCA, lactate levels are associated with resuscitation duration, post-cardiac arrest ischemia–reperfusion injury, and persistent circulatory failure after ROSC, and potentially serve as a useful prognostic indicator [8,9]. Moreover, serum albumin, a negative acute-phase reactant, reflects nutritional status and critical illness severity. Hypoalbuminemia is associated with systemic inflammation, increased capillary permeability, reduced hepatic synthesis, and adverse outcomes in critically ill and post-cardiac arrest patients [10,11]. Accordingly, the lactate-to-albumin ratio (LAR) has been proposed as a composite marker integrating hypoperfusion-related metabolic stress and illness severity reflected by serum albumin, and has been investigated as a prognostic tool in critically ill and post-cardiac arrest populations [12,13,14,15].

After ROSC, a systemic inflammatory response develops rapidly, and C-reactive protein (CRP) is one of the most commonly used circulating markers of this response [16]. The C-reactive protein-to-albumin ratio (CAR) has been proposed to provide superior prognostic value than that of CRP or albumin alone, as its components reflect illness severity and systemic inflammation. Several OHCA studies also report associations between higher CAR values and worse outcomes [17,18].

LAR and CAR may be clinically accessible and practical biomarkers because they can be calculated rapidly from routinely available laboratory tests without additional specialized assays or substantial cost. In addition, these ratio-based markers may partly overcome the physiological limitations of interpreting individual biomarkers in isolation by integrating complementary information on metabolic stress, systemic inflammation, and albumin-related physiological reserve. In previous OHCA studies, it has been suggested that LAR has prognostic value and may provide better predictive performance than lactate or albumin alone, whereas CAR has also been associated with mortality after cardiac arrest [13,15,17,18]. However, in most previous studies, these ratios were assessed separately or at a single time point, and direct comparisons using serial measurements remain limited. Given the dynamic nature of post-cardiac arrest syndrome, in this study, we aimed to evaluate the association between serial LAR and CAR measurements during the first 72 h after ROSC and in-hospital mortality in patients with OHCA.

## 2. Materials and Methods

### 2.1. Study Design and Population

This retrospective observational cohort study included patients with OHCA treated with targeted temperature management (TTM) at Chonnam National University Hospital from January 2022 to December 2025. Eligible participants were comatose adults aged ≥18 years after TTM completion. Patients were excluded if their TTM protocol was interrupted owing to inter-facility transfer or early mortality, or if essential blood samples were unavailable. The study protocol was approved by the Institutional Review Board of our institution, which formally waived the requirement for written informed consent owing to the retrospective review of medical records [14].

All comatose cardiac arrest survivors underwent TTM in accordance with institutional guidelines. A target body temperature of 33.0–36.0 °C was maintained for 24 h using an Arctic Sun^®^ feedback-controlled surface cooling device (Energy Transfer Pads^TM^; Medivance Corp., Louisville, CO, USA). Following the maintenance phase, controlled rewarming was performed at a rate of 0.25 °C/h until a core temperature of 36.5 °C was reached.

### 2.2. Data Collection and Primary Outcome

Baseline demographics, preexisting comorbidities, and arrest-specific variables, including witnessed collapse, bystander cardiopulmonary resuscitation (CPR), initial cardiac rhythm, arrest etiology, and collapse-to-ROSC interval, were abstracted from electronic medical records. On emergency department (ED) arrival, initial arterial blood gas parameters (partial pressure of oxygen [PaO_2_] and partial pressure of carbon dioxide [PaCO_2_]) and serum glucose levels were recorded. The Sequential Organ Failure Assessment (SOFA) score was calculated using data from the initial 24 h of admission. Serum lactate, albumin, and CRP levels were serially measured at four time points, namely, admission, 24, 48, and 72 h after ROSC, to assess dynamic metabolic and inflammatory profiles. These values were utilized to derive the LAR and CAR. Our primary endpoint was in-hospital mortality.

### 2.3. Statistical Analysis

A complete-case analysis was performed; patients with any missing laboratory measurements at any of the predefined time points were excluded, and no data imputation was applied. Categorical variables are summarized as frequencies (percentages) and compared using the chi-squared test with continuity correction for 2 × 2 tables. Continuous variables were assessed for normality using the Shapiro–Wilk test. Owing to the asymmetric distribution of the data, continuous variables are presented as medians with interquartile ranges (IQRs) and analyzed using the Mann–Whitney U test. Multivariate logistic regression was performed to identify independent predictors of in-hospital mortality. Candidate variables with a *p*-value < 0.20 (hypertension, witnessed collapse, bystander CPR, and cardiac etiology) in univariate analyses were selected for the final adjusted model (Supplementary Table S1), along with shockable rhythm and time from collapse to ROSC. To strictly control for potential confounding factors and prevent the overestimation of independent associations in proportions, the final adjusted model was expanded to include clinically relevant prognostic factors that showed significant differences in baseline comparisons between survivors and non-survivors. Finally, age, shockable rhythm, time from collapse to ROSC, PaCO_2_ level, and the SOFA score were included. LAR and CAR at each time point were included in the final model. To account for multiple comparisons arising from the serial measurements of LAR and CAR across four time points (total of 8 analyses), the Holm–Bonferroni correction was applied to adjust the *p*-values of the multivariable logistic regression models. Adjusted *p*-values < 0.05 were considered statistically significant. The results are expressed as odds ratios (ORs) with 95% confidence intervals (CIs). The predictive performance of LAR and CAR was evaluated using the area under the receiver operating characteristic curve (AUC). Additionally, a post hoc power analysis was performed to evaluate the adequacy of the sample size based on the AUC for predicting in-hospital mortality, with a two-sided alpha level of 0.05. Statistical analyses were performed utilizing PASW Statistics 18.0 (SPSS Inc., Chicago, IL, USA) and MedCalc 19.0 (MedCalc Software, Ostend, Belgium), with statistical significance set at *p* < 0.05.

## 3. Results

### 3.1. Patient Characteristics

**Figure 1:**
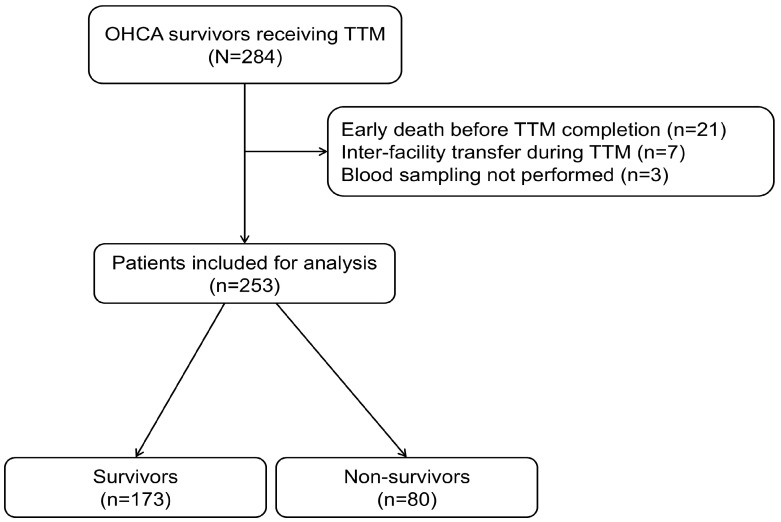
Schematic diagram showing the number of patients in the present study. OHCA, out-of-hospital cardiac arrest; TTM, target temperature management.

Of the 284 patients with OHCA treated with TTM, 31 were excluded due to early mortality (*n* = 21), inter-facility transfer (*n* = 7), or missing serial laboratory data (*n* = 3). Therefore, 253 patients were included in the final analysis (Figure 1). Of these, 173 survived to hospital discharge, and 80 died in hospital, corresponding to an in-hospital mortality rate of 31.6%. The median age was 61 years, and 195 patients (77.1%) were men. Overall, 168 patients (66.4%) had a witnessed arrest, and 167 (66.0%) received bystander CPR. The initial arrest rhythm was shockable in 104 patients (41.1%), and 138 (54.5%) had a cardiac etiology of arrest. The median duration from collapse to ROSC was 28.0 min (19.0–41.0).

Table 1 shows baseline characteristics between survivors and non-survivors. Survivors had a higher proportion of initial shockable rhythm and a cardiac etiology of arrest compared with non-survivors. Survivors also had a shorter time from collapse to ROSC, lower PaCO_2_ after ROSC, and lower SOFA scores (Table 1).

### 3.2. Association of LAR and CAR with In-Hospital Mortality

Table 2 illustrates that LAR and CAR were significantly higher in non-survivors than in survivors at admission, and at 24, 48, and 72 h after ROSC. After adjustment for potential confounders and applying the Holm–Bonferroni correction, LAR maintained its independent association with in-hospital mortality at all assessed time points (Table 3). However, CAR was independently associated with in-hospital mortality only at 72 h after ROSC (adjusted *p* = 0.008), whereas it showed no significant association at admission (adjusted *p* = 0.087) and 24 h (adjusted *p* = 0.306), and demonstrated a borderline non-significant trend at 48 h (adjusted *p* = 0.064).

### 3.3. Prognostic Performances of the LAR and CAR for In-Hospital Mortality

The AUCs of LAR for predicting in-hospital mortality at admission, 24, 48, and 72 h after ROSC were 0.715 (0.655–0.770), 0.720 (0.660–0.774), 0.734 (0.675–0.788), and 0.702 (0.635–0.752), respectively. The AUCs of CAR at the corresponding time points were 0.640 (0.578–0.699), 0.634 (0.571–0.693), 0.651 (0.589–0.710), and 0.690 (0.629–0.746), respectively (Table 4). In the post hoc power analysis based on our sample size (*n* = 253; 173 survivors and 80 non-survivors) and an alpha level of 0.05, the statistical power to detect the discriminatory performance of LAR was >99.9% across all time points. For CAR, the statistical power was 91.2% at 24 h, 98.4% at admission, and >99.9% at 48 h and 72 h, confirming that the study sample size was sufficient to provide robust statistical conclusions.

### 3.4. Sensitivity Analysis Excluding Patients with Renal Impairment and Chronic Liver Disease

To further verify the robustness of our findings and minimize the potential confounding effects of preexisting organ dysfunctions on the biomarkers, we performed a sensitivity analysis by excluding patients with renal impairment and chronic liver disease (Supplementary Table S2). After adjustment for potential confounders and applying the Holm–Bonferroni correction, LAR consistently maintained independent association with in-hospital mortality across all assessed time points (all *p* < 0.001). In contrast, CAR was independently associated with in-hospital mortality only at 72 h after ROSC (adjusted *p* = 0.032).

## 4. Discussion

In this retrospective cohort of post-ROSC OHCA patients, LAR and CAR levels measured at admission and during the first 72 h were consistently higher in non-survivors than in survivors. In multivariable analyses, LAR remained independently associated with in-hospital mortality at all assessed time points, whereas CAR retained its independent association with mortality only at 72 h post-ROSC after Holm–Bonferroni correction. In AUC analysis, LAR revealed modest performance, whereas CAR showed poor performance for predicting in-hospital mortality.

Studies show that higher LAR values are associated with poor outcomes in patients with OHCA [12,13,15]. Among these studies, Kokulu et al. further show that LAR measured within 10 min of ED arrival has better predictive performance for survival to hospital discharge than lactate or albumin alone [13]. Nakada et al., in a nationwide multicenter study, likewise show that hospital-arrival LAR has better predictive value than either lactate or albumin alone for favorable neurological outcomes after OHCA [15]. These findings may be explained by the complementary pathophysiological information provided by lactate and albumin. Although lactate primarily reflects the severity of ischemia–reperfusion injury and metabolic stress, its interpretation as a single biomarker is limited. This is because lactate levels can also be influenced by impaired hepatic and renal clearance, exogenous catecholamine administration, and seizures. By incorporating albumin, which may reflect systemic inflammation, capillary leak, and overall physiological reserve after ROSC, LAR may better capture the complex pathophysiology of post-cardiac arrest syndrome [3,8,9,10,11]. In this context, our findings suggest that LAR may serve as a clinically useful prognostic marker in patients with OHCA.

Differences between studies should be considered when interpreting absolute LAR values and their prognostic performance. Compared with our cohort, Kong et al. reported higher LAR values in non-survivors and survivors. LAR values in non-survivors were 4.91 in the Kong cohort and 3.57 in our cohort, while in survivors, they were 2.73 and 1.79, respectively. This difference may partly reflect the longer collapse-to-ROSC time in the Kong cohort than in our cohort (37.3 vs. 28.0 min) [12]. In the study by Kokulu et al., the outcome distribution differed substantially from that in our cohort, with a much higher proportion of non-survivors (82.1% vs. 31.6%) [13]. Although detailed causes of death were not reported, differences in baseline patient characteristics, resuscitation-related factors, and institutional end-of-life practices, including withdrawal of life-sustaining treatment, may have contributed to this disparity. These factors should be considered when interpreting between-study differences [13]. Moreover, because differences in post-resuscitation care protocols, including TTM target temperature, cooling duration, rewarming rate, and hemodynamic management, may influence lactate clearance and albumin concentrations, direct comparisons of absolute LAR values between previous studies and our cohort should be interpreted with caution [12,13]. In the nationwide study by Nakada et al., LAR was assessed using the first post-arrival measurement obtained in routine clinical practice [15]. In contrast, our study focused on in-hospital mortality and evaluated LAR serially at predefined time points over the first 72 h after ROSC. This serial approach allowed us to assess the time-specific prognostic performance of LAR and its temporal pattern after ROSC. In our cohort, the difference in LAR values between survivors and non-survivors was greater at admission than at later time points and decreased over time. This finding may reflect a progressive decline in LAR values in both groups as physiological status partially stabilized after ROSC. Nevertheless, the discriminatory performance of LAR was relatively maintained from admission to 48 h but decreased at 72 h, suggesting that it retains prognostic value during the early post-resuscitation period despite decreasing values over time.

Consistent with previous findings, higher CAR values in our cohort were associated with worse outcomes after OHCA [17,18]. However, despite this association, CAR showed poor discriminatory performance for predicting in-hospital mortality across all assessed time points in our study. Kim et al. also performed serial CAR measurements at admission and at 24, 48, and 72 h after cardiac arrest [18]. Consistent with our findings, the discriminatory performance of CAR was low at admission but increased over time [18]. This temporal pattern is broadly consistent with our results and may reflect the delayed kinetics of CRP after ROSC. CRP synthesis begins several hours after an inflammatory stimulus, with serum levels typically rising within 4–6 h and peaking at approximately 36–48 h rather than immediately after the insult [19,20]. Accordingly, early CAR values may incompletely reflect the evolving inflammatory burden after ROSC. Conversely, later measurements may better capture the progression of post-cardiac arrest systemic inflammation and the additional contribution of infectious complications. Albumin, the denominator of CAR, may also be influenced by baseline physiological reserve, inflammation-related capillary leakage, altered hepatic synthesis, and fluid administration. Therefore, the limited early performance of CAR may reflect not only the delayed rise in CRP but also a temporal and biological mismatch between CRP and albumin during the early post–cardiac arrest phase [3,11,21,22].

The limited discriminatory performance of CAR is also consistent with findings from previous studies of critically ill non-OHCA populations [23,24,25]. Park et al. report that CAR has poor discriminatory performance for 28-day mortality in medical ICU patients (AUC, 0.594), although its performance is slightly higher than that of CRP alone (AUC, 0.567) [23]. Similarly, in ICU patients with traumatic brain injury, CAR was associated with ICU mortality, but its discriminatory performance was poor to modest (AUC, 0.642), with sensitivity and specificity of 61.0% and 60.1%, respectively [24]. In patients with acute stroke, CAR showed good discrimination for in-hospital mortality, but its performance did not differ significantly from that of CRP alone (AUC, 0.824 vs. 0.820; *p* = 0.287) [25]. This suggests that the additional prognostic value of albumin within CAR may be limited in some critically ill populations [23,25]. Thus, CAR may reflect illness severity and inflammatory burden, but its utility as a standalone mortality predictor may be limited.

Taken together, our findings suggest that LAR may be more clinically informative than CAR in post-ROSC OHCA patients, given its more consistent association with in-hospital mortality and modestly better discriminatory performance. In contrast, CAR showed poor discriminatory performance across all assessed time points, suggesting limited clinical utility as a standalone prognostic marker in this cohort. Therefore, if one of these ratios is considered as an adjunctive laboratory marker, LAR appears preferable to CAR. Importantly, our study was not designed to compare the sensitivity of LAR or CAR with prehospital or conventional prognostic indicators; therefore, these ratios should be viewed as complementary markers rather than superior alternatives. Neither LAR nor CAR should be interpreted as a standalone prognostic or clinical decision-making tool, or as a substitute for established clinical indicators and multimodal post-cardiac arrest prognostication.

## 5. Limitations

This study has some limitations. First, this was a retrospective single-center study, which may limit the generalizability of our findings to other clinical settings and introduce potential selection bias. In particular, our cohort had a relatively high survival rate and a substantial proportion of patients with an initial shockable rhythm; therefore, external validation in independent multicenter cohorts is required. Second, the interpretation of serial biomarker measurements may have been affected by survivor bias, because these measurements could only be obtained from patients who survived long enough to complete the scheduled laboratory assessments. Third, although we adjusted for key resuscitation-related factors, including initial shockable rhythm and time to ROSC, residual confounding from unmeasured variables cannot be excluded. Fourth, causes of in-hospital death and infection-related factors were not systematically collected or adjudicated in this retrospective study. Therefore, the pathophysiological mechanisms underlying the associations between LAR, CAR, and mortality should be interpreted cautiously, particularly because elevated CAR may reflect both sterile post-cardiac arrest inflammation and infectious complications. Finally, in this study, we did not evaluate whether LAR or CAR provides incremental prognostic value beyond individual component biomarkers or established post-cardiac arrest prognostic models. Therefore, these ratios should be interpreted as adjunctive laboratory markers rather than standalone tools for prognostication or treatment decision-making. Future prospective multicenter studies should validate predefined cut-off values at standardized sampling time points and assess whether serial LAR adds clinically meaningful prognostic information beyond established clinical predictors and prognostic models.

## 6. Conclusions

In this retrospective cohort of post-ROSC OHCA patients, recurrent assessments of LAR during the early post-resuscitation period consistently correlated with in-hospital mortality and showed modest discriminatory performance. In contrast, CAR was associated with mortality at 72 h after ROSC but showed poor discriminatory performance, suggesting limited clinical utility as a standalone prognostic marker. Further prospective multicenter studies are needed to validate predefined LAR cut-off values at standardized sampling time points and to determine whether serial LAR adds incremental prognostic value beyond established post-cardiac-arrest prognostic models.

## Figures and Tables

**Table 1:** Comparisons of baseline characteristics according to in-hospital mortality.

Variables	Total (*n* = 253)	Survivors (*n* = 173)	Non-Survivors (*n* = 80)	*p*-Value
Demographics				
Age, years	61.0 (49.0–69.5)	62.0 (49.5–69.0)	61.0 (48.3–70.0)	0.661
Male, n (%)	195 (77.1)	136 (78.6)	59 (73.8)	0.487
Preexisting illness, n (%)				
Coronary artery disease	26 (10.3)	18 (10.4)	8 (10.0)	1.000
Congestive heart failure	13 (5.1)	8 (4.6)	5 (6.3)	0.812
Hypertension	121 (47.8)	88 (50.9)	33 (41.3)	0.198
Diabetes	75 (29.6)	48 (27.7)	27 (33.8)	0.410
Chronic lung disease	21 (8.3)	12 (6.9)	9 (11.3)	0.362
Renal impairment	29 (11.5)	19 (11.0)	10 (12.5)	0.889
Cerebrovascular accident	16 (6.3)	10 (5.8)	6 (7.5)	0.807
Chronic liver disease	8 (3.2)	3 (1.7)	5 (6.3)	0.128
Cardiac arrest characteristics				
Witnessed collapse, n (%)	168 (66.4)	121 (69.9)	47 (58.8)	0.108
Bystander CPR, n (%)	167 (66.0)	120 (69.4)	47 (58.8)	0.130
Shockable rhythm, n (%)	104 (41.1)	87 (50.3)	17 (21.3)	<0.001
Cardiac etiology, n (%)	138 (54.5)	108 (62.4)	30 (37.5)	<0.001
Time from collapse to ROSC, min	28.0 (19.0–41.0)	25.0 (18.0–36.0)	40.0 (26.0–50.0)	<0.001
Clinical characteristics after ROSC				
Glucose, mg/dL	264 (191–318)	254 (190–316)	271 (199–321)	0.413
PaO_2_, mmHg	184.0 (98.5–274.0)	189.0 (99.0–275.0)	172.0 (98.0–273.5)	0.653
PaCO_2_, mmHg	43.0 (35.5–63.0)	41.0 (35.0–58.8)	53.5 (38.9–68.6)	0.025
SOFA score	11 (10–13)	11 (9–13)	12 (10–14)	<0.001

CPR, cardiopulmonary resuscitation; ROSC, restoration of spontaneous circulation; PaO_2_, partial pressure of oxygen; PaCO_2_, partial pressure of carbon dioxide; SOFA, Sequential Organ Failure Assessment.

**Table 2:** Comparisons of LAR and CAR according to in-hospital mortality.

Variables	Total (*n* = 253)	Survivors (*n* = 173)	Non-Survivors (*n* = 80)	*p*-Value
LAR at admission	2.16 (1.35–3.45)	1.79 (1.22–2.80)	3.57 (1.78–4.97)	<0.001
LAR at 24 h	0.58 (0.36–0.97)	0.47 (0.33–0.78)	0.90 (0.52–1.49)	<0.001
LAR at 48 h	0.46 (0.33–0.84)	0.42 (0.31–0.61)	0.79 (0.45–1.51)	<0.001
LAR at 72 h	0.40 (0.30–0.66)	0.36 (0.29–0.53)	0.55 (0.38–1.04)	<0.001
CAR at admission	0.04 (0.01–0.19)	0.03 (0.01–0.10)	0.07 (0.01–1.12)	0.006
CAR at 24 h	2.42 (1.45–3.64)	2.06 (1.25–3.33)	3.01 (1.76–4.07)	<0.001
CAR at 48 h	5.46 (3.41–7.24)	4.98 (3.10–6.70)	6.32 (4.57–8.67)	<0.001
CAR at 72 h	5.10 (3.49–7.67)	4.69 (3.27–6.70)	7.19 (4.73–10.80)	<0.001

LAR, lactate-to-albumin ratio; CAR, C-reactive protein-to-albumin ratio. LAR was calculated as serum lactate (mmol/L) divided by serum albumin (g/dL). CAR was calculated as serum C-reactive protein (mg/dL) divided by serum albumin (g/dL). Values are presented as medians with interquartile ranges.

**Table 3:** Multivariate logistic regression analysis of LAR and CAR for in-hospital mortality.

Variables	Adjusted OR (95% CI) ^a^	*p*-Value
LAR at admission	1.465 (1.241–1.730)	<0.001
LAR at 24 h	2.795 (1.670–4.676)	<0.001
LAR at 48 h	2.883 (1.695–4.904)	<0.001
LAR at 72 h	3.495 (1.708–7.153)	<0.001
CAR at admission	1.332 (1.029–1.725)	0.087
CAR at 24 h	1.090 (0.924–1.286)	0.306
CAR at 48 h	1.122 (1.010–1.246)	0.064
CAR at 72 h	1.146 (1.052–1.247)	0.008

LAR, lactate-to-albumin ratio; CAR, C-reactive protein-to-albumin ratio; OR, odds ratio; CI, confidence interval; PaCO_2_, partial pressure of carbon dioxide; SOFA, Sequential Organ Failure Assessment. Each variable was individually entered into the final model and analyzed separately. *p*-Values were adjusted using the Holm–Bonferroni correction for multiple comparisons. ^a^ Adjusted for age, shockable rhythm, time from collapse to return of spontaneous circulation, PaCO_2_ level, and the SOFA score.

**Table 4:** AUC analysis of LAR and CAR for in-hospital mortality.

Variable	AUC (95% CI)	Cut-Off Value	Sensitivity (95% CI)	Specificity (95% CI)
LAR at admission	0.715 (0.655–0.770)	>3.4	52.5 (41.0–63.8)	87.9 (82.0–92.3)
LAR at 24 h	0.720 (0.660–0.774)	>1.0	45.0 (33.8–56.5)	89.6 (84.1–93.7)
LAR at 48 h	0.734 (0.675–0.788)	>0.5	70.0 (58.7–79.7)	71.1 (63.7–77.7)
LAR at 72 h	0.702 (0.635–0.752)	>0.8	35.0 (24.7–46.5)	93.1 (88.2–96.4)
CAR at admission	0.640 (0.578–0.699)	>0.1	42.5 (31.5–54.1)	80.9 (74.3–86.5)
CAR at 24 h	0.634 (0.571–0.693)	>2.1	70.0 (58.7–79.7)	52.6 (44.9–60.2)
CAR at 48 h	0.651 (0.589–0.710)	>4.3	80.0 (69.6–88.1)	43.4 (35.9–51.1)
CAR at 72 h	0.690 (0.629–0.746)	>6.8	55.0 (43.5–66.2)	76.3 (69.3–82.4)

AUC, area under the curve; LAR, lactate-to-albumin ratio; CAR, C-reactive protein-to-albumin ratio; OR, odds ratio; CI, confidence interval.

## Data Availability

The data presented in this study are available upon request from the corresponding author. The data are not publicly available due to personal protection.

## Supplementary Materials

The following supporting information can be downloaded at: https://www.mdpi.com/article/10.3390/jcm15134851/s1, Table S1: Multivariate logistic regression analysis for in-hospital mortality; Table S2. Multivariate logistic regression analysis of LAR and CAR for in-hospital mortality, excluding patients with renal impairment and chronic liver disease.

## Abbreviations

The following abbreviations are used in this manuscript:

LAR	Lactate-to-albumin ratio
CAR	C-reactive protein-to-albumin ratio
ROSC	Return of spontaneous circulation
OHCA	Out-of-hospital cardiac arrest
AUC	Area under the curve
CRP	C-reactive protein
TTM	Targeted temperature management
CPR	Cardiopulmonary resuscitation
ED	Emergency department
PaO_2_	Partial pressure of oxygen
PaCO_2_	Partial pressure of carbon dioxide
SOFA	Sequential Organ Failure Assessment
IQR	Interquartile ranges
OR	Odds ratio
CI	Confidence interval
