# Supplementary material for: Association of Serial Lactate-to-Albumin and C-Reactive Protein-to-Albumin Ratios with In-Hospital Mortality After Out-of-Hospital Cardiac Arrest
Source: J Clin Med. 2026 Jun 23;15(13):4851. doi: 10.3390/jcm15134851 (PMC13361076; doi:10.3390/jcm15134851)
Supplement: Supplementary file 1 [file jcm-15-04851-s001.zip › jcm-4363293-supplementary.pdf]

Supplementary Table S1. Multivariate logistic regression analysis for in-hospital mortality.

**Variables**	**Adjusted OR (95% CI)**	***p*-Value**
Hypertension	0.610 (0.329–1.130)	0.116
Witness of collapse	0.880 (0.458–1.694)	0.703
Bystander CPR	0.676 (0.364–1.257)	0.676
Shockable rhythm	0.239 (0.124–0.461)	< 0.001
Cardiac etiology	0.665 (0.303–1.458)	0.308
Time to ROSC, min	1.037 (1.018–1.056)	< 0.001
$PaCO_2$, mmHg	0.993 (0.979–1.008)	0.364
SOFA score	1.107 (0.994–1.233)	0.065

OR, odds ratio; CI, confidence interval; CPR, cardiopulmonary resuscitation; ROSC, restoration of spontaneous circulation; $PaCO_2$, partial pressure of carbon dioxide; SOFA, Sequential Organ Failure Assessment.

Supplementary Table S2. Multivariate logistic regression analysis of LAR and CAR for in-hospital mortality, excluding patients with renal impairment and chronic liver disease.

**Variables**	**Adjusted OR (95% CI)** [a]	***p*-Value**
LAR at admission	1.458 (1.222–1.740)	<0.001
LAR at 24 hours after ROSC	2.798 (1.569–4.991)	<0.001
LAR at 48 hours after ROSC	2.665 (1.512–4.696)	<0.001
LAR at 72 hours after ROSC	4.457 (1.820–10.915)	<0.001
CAR at admission	1.244 (0.974–1.589)	0.240
CAR at 24 hours after ROSC	0.978 (0.811–1.179)	0.813
CAR at 48 hours after ROSC	1.078 (0.955–1.216)	0.446
CAR at 72 hours after ROSC	1.141 (1.035–1.258)	0.032

LAR, lactate-to-albumin ratio; CAR, C-Reactive Protein-to-albumin ratio; OR, odds ratio; CI, confidence interval; PaCO2, partial pressure of carbon dioxide; SOFA, Sequential Organ Failure Assessment.

Each variable was individually entered into the final model and analyzed separately.

p-Values were adjusted using the Holm–Bonferroni correction for multiple comparisons.

[a]Adjusted for age, shockable rhythm, time from collapse to return of spontaneous circula-tion, PaCO2 level, and the SOFA score.
